# Multiplexed CRISPR base editing enables pulse-activated irreversible biocontainment of engineered bacteria

**DOI:** 10.1093/nar/gkag422

**Published:** 2026-05-04

**Authors:** Sung Won Cho, TaeHyun Kim, Jina Yang, Gibyuck Byun, Sang Woo Seo

**Affiliations:** Department of Chemical and Biological Engineering, Seoul National University, 1 Gwanak-ro, Gwanak-Gu, Seoul 08826, Republic of Korea; Department of Chemical and Biological Engineering, Seoul National University, 1 Gwanak-ro, Gwanak-Gu, Seoul 08826, Republic of Korea; Department of Chemical Engineering, Jeju National University, 102 Jejudaehak-ro, Jeju-si, Jeju-do 63243, South Korea; Department of Chemical and Biological Engineering, Seoul National University, 1 Gwanak-ro, Gwanak-Gu, Seoul 08826, Republic of Korea; Department of Chemical and Biological Engineering, Seoul National University, 1 Gwanak-ro, Gwanak-Gu, Seoul 08826, Republic of Korea; Institute of Chemical Processes, Seoul National University, 1 Gwanak-ro, Gwanak-gu, Seoul 08826, South Korea; Bio-MAX Institute, Seoul National University, 1 Gwanak-ro, Gwanak-gu, Seoul 08826, South Korea; Institute of Bio Engineering, Seoul National University, 1 Gwanak-ro, Gwanak-gu, Seoul 08826, South Korea; Interdisciplinary Program in Bioengineering, Seoul National University, 1 Gwanak-ro, Gwanak-gu, Seoul 08826, South Korea

## Abstract

The environmental and therapeutic application of genetically engineered microorganisms necessitates the development of robust, irreversible biocontainment systems. In this study, we present an eEGM (editing-driven essential gene multiplex inactivation) module that utilizes CRISPR-mediated cytidine base editing to induce permanent self-killing via a single transient induction. By targeting the start codons of essential genes, we achieved an irreversible translational blockade that avoids the fitness costs associated with basal toxicity in nuclease-based systems. Multiplexed targeting of non-redundant essential loci (*holA, ftsB*, and *dfp*) yielded escape frequencies at or below the NIH guideline criterion (10^−8^) within 1 h of pulse induction. Furthermore, the eEGM system exhibited robust functional orthogonality and portability across laboratory, industrial, and therapeutic *Escherichia coli* strains, including MG1655, W3110, and Nissle 1917, without detectable interference with heterologous protein expression. This work establishes base editing as a cleavage-free CRISPR effector for pulse-activated, irreversible biocontainment and provides a practical framework for safer deployment of engineered microbes.

## Graphical Abstract

**Graphical Abstract:**
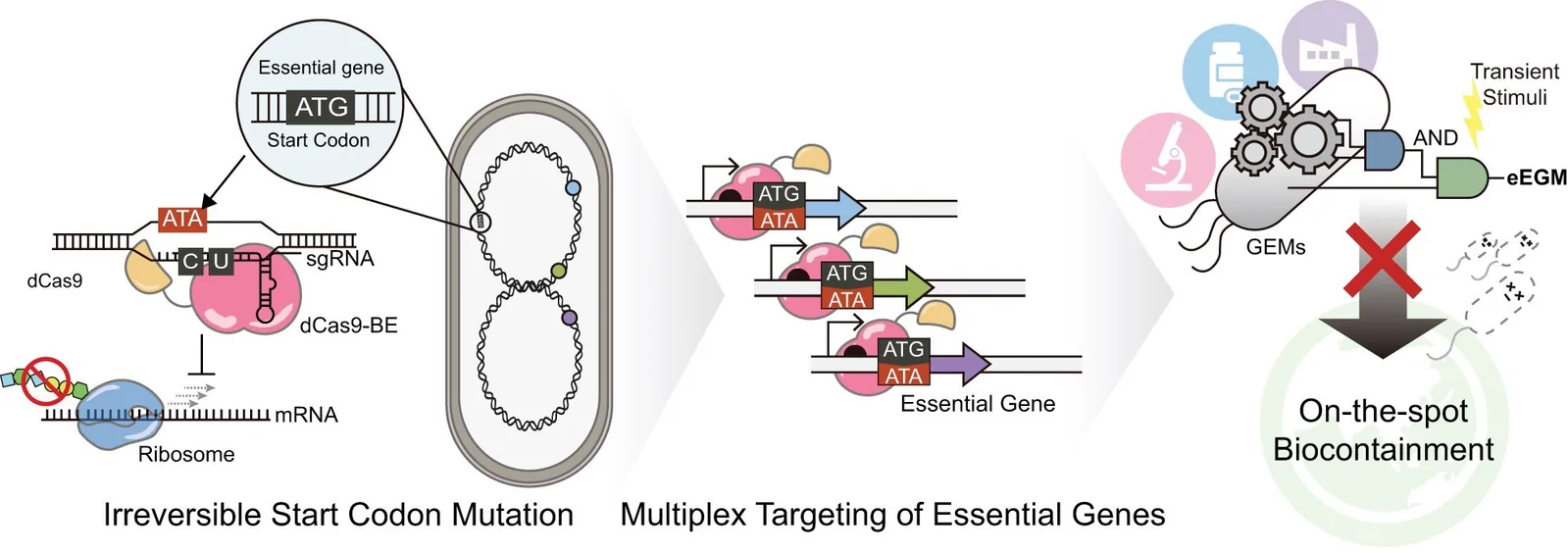

## Introduction

Biocontainment is a foundational requirement in the synthetic biology field for the safe deployment of engineered microorganisms (GEMs) in industrial, environmental, and therapeutic applications beyond strictly contained laboratory environments [1–5]. Early genetic containment strategies, such as toxin–antitoxin kill switches and synthetic auxotrophs, have established important proof-of-concept safeguards by demonstrating the feasibility of genetically encoded containment [6–9]. These approaches highlight key principles for limiting microbial persistence and provide a critical foundation for subsequent biocontainment technologies. At the same time, different strategies are optimized for distinct application contexts and therefore introduce trade-offs in terms of operational simplicity, portability, and deployment scope [10–13]. Even advanced orthogonal auxotrophic systems based on noncanonical amino acids, which substantially reduce evolutionary escape, require extensive host-specific chromosomal engineering and controlled supplementation, posing challenges for broad applicability across strains and use cases [9, 10, 14, 15]. To address these systemic challenges, several studies have emphasized that robust biocontainment is inherently a system-level problem, motivating the exploration of complementary strategies that can expand the range of genetic parts and regulatory thresholds without relying on toxin production or extensive genome recoding [11, 16, 17].

CRISPR-Cas9-based biocontainment has emerged as a powerful extension of classical safeguards by enabling programmable, toxin-free loss of cellular viability through direct targeting of essential genetic functions [3, 18–20]. Nuclease-active Cas9 provides lethality through DNA cleavage, which can be implemented across diverse species [21–23], but faces a fundamental trade-off between efficacy and host fitness costs arising from off-target cleavage [19, 21]. Such compensation necessitates chromosomal integration or multilayered, complex regulatory architectures to prevent unintended cell death [18, 24], which undermine the operational simplicity and portability demanded in the field. In contrast, nuclease-deficient dCas9 (CRISPRi) eliminates such fitness cost but provides only transient, reversible repression that is lost upon the cessation of induction [25–27]. This gap between irreversible but toxic cleavage-based strategies and non-toxic but reversible binding-based approaches motivates the exploration of alternative CRISPR modalities. Emerging fusion-based tools, such as base editors (BEs), offer a unique solution by enabling permanent nucleotide conversion without inducing double-strand breaks [28–32]. By leveraging dCas9-driven BE, genetic safeguards can achieve irreversible outcomes with minimal basal burden and precise temporal control.

Here, we introduce a biocontainment strategy that leverages programmable BE to permanently disrupt the start codons of essential genes. Through *in silico* screening and *in vivo* validation of 15 single guide RNAs (sgRNAs), we identify three highly efficient candidates. By incorporating additional regulation, we develop an editing-based essential gene targeting (eEG) module. The eEG system achieves robust, irreversible repression following transient induction, overcoming the reversibility of the dCas9-mediated interference (iEG) system. We further develop the system to target multiple essential genes simultaneously, enabling the multiplexed system (eEGM) to surpass the NIH guideline criterion (10^−8^) within 1 h of induction, while maintaining growth kinetics indistinguishable from wild-type controls. Our eEGM system demonstrates long-term stability over 10 serial passages and functional portability across multiple *Escherichia coli* derivatives without interfering with heterologous gene expression. Our study establishes BE as an operationally efficient, nuclease-independent effector for stringent and portable microbial biocontainment.

## Materials and methods

### Computational screening and design of sgRNAs

Essential gene targets were computationally selected to enable programmable CRISPR-based biocontainment. A total of 302 essential genes were retrieved from the PEC Essential Gene Database (Profiling of *E. coli* Chromosomes) [33]. For each gene, the coding sequence and upstream region were extracted from the *E. coli* MG1655 reference genome to generate a searchable sequence library. Spacer candidates (20 nt) were screened according to cytidine base-editing criteria, requiring a cytidine within the editing window positioned 16–19 bp upstream of a canonical NGG PAM site [30]. Custom Python scripts were used to identify protospacers meeting these positional constraints, and spacers containing additional cytidines or guanines within the window that could result in additional editing were included, resulting in 11 candidates. Candidate protospacers were evaluated for predicted on-target efficiency using established scoring metrics implemented in the CRISPOR web tool to prioritize guides with higher expected editing activity [34]. Guides with higher predicted activity and without sequence features associated with reduced performance were retained for experimental validation. The complete list of target essential genes and spacer sequences is provided in Supplementary Table S1.

### Bacterial strains and culture conditions

The bacterial strains used in this study are listed in Supplementary Table S2. All culture experiments were performed in Luria-Bertani (BD Bioscience) medium supplemented with appropriate antibiotics at 37°C in a rotary shaker (250 rpm) to ensure adequate oxygen transfer. Antibiotics (all from Golden Biotechnology, Taipei, Taiwan) were added at the following final concentrations unless otherwise specified: chloramphenicol, 34 μg/ml; spectinomycin, 50 μg/ml; kanamycin, 50 μg/ml. Cultures were incubated at 37°C with shaking at 250 rpm in 14 ml round-bottom test tubes. Temperature was reduced to 25°C after induction for all biocontainment-related assays.

### Reagents and oligonucleotides

All oligonucleotides synthesized by Bionics (Korea) are listed in Supplementary Table S3. Q5 High-Fidelity DNA Polymerase, restriction enzymes (BsaI, BamHI, AvrII, AatII, SphI, and SpeI), and Quick Ligase were purchased from New England Biolabs (NEB, Ipswich, MA, USA). Gibson Assembly Master Mix was obtained from Codex. Plasmid DNA was extracted using the Exprep™ Plasmid SV kit (GeneAll), and polymerase chain reaction (PCR) amplicons were purified using the Zymo DNA Clean & Concentrator™ kit (Zymo Research). Anhydrotetracycline (aTc) and arabinose were purchased from Gold Biotechnology. Theophylline and rhamnose were obtained from Sigma–Aldrich. All other general reagents were purchased from Sigma–Aldrich unless otherwise noted.

### Plasmid construction

Single-sgRNA expression plasmids were generated by inserting annealed spacer oligonucleotides into a BsaI-digested sgRNA backbone plasmid driven by a rhamnose-inducible promoter (pRha). Spacer oligonucleotides were synthesized as complementary single-stranded oligos, heated to 95 °C for 5 min, and gradually cooled to room temperature to allow annealing. Double-stranded inserts were ligated into the digested backbone using Quick Ligase (New England Biolabs), and ligation mixtures were transformed into NEB 10-beta chemically competent *E. coli*. Multi-sgRNA expression plasmids were generated by concatenating three individual pRha-driven sgRNA transcription units using unique nucleotide sequence linkers, followed by BsaI restriction–ligation into the same sgRNA backbone. All sgRNA expression plasmids carried spectinomycin resistance.

CRISPR-Cas9 driven BE and knockdown plasmids were constructed from the parental pdCas9 backbone digested with BamHI and AvrII. Knockdown variants were assembled from two fragments: a fragment encoding the C-terminal region of dCas9 fused to an LVA degradation tag and a transcriptional terminator, and a single- or multi-sgRNA cassette excised from the sgRNA expression plasmids. BE variants were assembled from four fragments: a C-terminal dCas9 homology region, a cytidine deaminase coding sequence, a transcriptional terminator, and a single- or multi-sgRNA cassette. All constructs carrying chloramphenicol resistance gene were introduced into NEB 10-beta cells by heat-shock transformation. Theophylline-responsive riboswitch variants were constructed by digesting dCas9–LVA and dCas9–CDA plasmids with AatII and SphI, followed by Gibson Assembly with PCR fragments containing the riboswitch sequence and homology overhangs. Both single- and multi-sgRNA configurations were prepared. For enhanced induction, an *RhaS*-integrated BE plasmid was generated by digesting pTet_switch_dCas_CDA_multi with SpeI and ligating the SpeI-digested *RhaS* coding sequence PCR-amplified from *E. coli* MG1655 genomic DNA.

### Plasmid transformation and strain construction

Plasmids were introduced into electrocompetent *E. coli* by electroporation (Bio-Rad MicroPulser). For the initial sgRNA screening assays, *E. coli* MG1655 was co-transformed with pdCas9 and the corresponding pCDF-sgRNA#, sgRNA expression plasmid. Co-transformants were selected on LB agar supplemented with chloramphenicol (34 μg/ml) and spectinomycin (50 μg/ml) and cultured in LB broth containing the same antibiotics for plasmid maintenance. For the subsequent single-plasmid systems, including multiplex base-editing biocontainment assays, dCas9–LVA or dCas9–CDA plasmids with individual sgRNAs were transformed and selected with chloramphenicol (34 μg/ml) only.

### Time-course viability assay

Overnight cultures were grown in LB medium containing the appropriate antibiotics at 37°C with shaking at 250 rpm. The following day, individual cultures were diluted 1:100 into fresh LB medium with appropriate antibiotics and incubated until reaching an OD_600_ of 1.0, corresponding to a biomass level sufficient for induction. Three induction durations were tested in parallel: 1, 4, and 16 h (overnight). For sgRNA screening assays, gene expression was induced with anhydrotetracycline (200 ng/ml) and rhamnose (0.2% w/v). For assays using the complete single-sgRNA systems, the same combination was used, with additional theophylline (2 mM) when a theophylline-responsive riboswitch was integrated. These inducer concentrations were empirically determined based on previous studies to ensure sufficient activation of the regulatory circuits [35–37]. For multi-sgRNA systems, higher induction levels were applied: anhydrotetracycline (400 ng/ml), rhamnose (0.5% w/v), and theophylline (5 mM). Following inducer addition, cultures were immediately shifted to 25°C to simulate conditions relevant for cell disposal scenarios rather than active growth.

At each designated time point, aliquots were taken for CFU determination. For induced samples, 1 ml of culture was harvested by centrifugation and resuspended in 15 μl of fresh medium, and the concentrated suspension was subsequently serially diluted 1:5 across eight steps. This approach enabled the assessment of the entire viable population under induced conditions. For non-induced controls, cultures were initially diluted 1:100 and subsequently subjected to 10-fold serial dilutions across eight steps. From each dilution, 15 μl was spotted in triplicate onto LB agar plates. Induced samples were serially diluted 1:5 across eight steps to enable finer quantification of CFUs, whereas non-induced controls were initially diluted 1:100 and subsequently serially diluted 1:10 across eight steps. From each dilution, 15 μl was spotted in triplicate onto LB agar plates containing the appropriate antibiotics for plasmid maintenance. Plates were incubated at 25°C overnight, and CFUs were counted manually. The presence of at least one visible colony was considered indicative of survival. Based on the plated volume (15 μl) and dilution schemes, the effective detectable ranges were estimated to be ~1 × 10^0^ to 3.9 × 10^5^ CFU/ml for induced samples and 6.7 × 10^3^ to 6.7 × 10^11^ CFU/ml for non-induced samples. Accordingly, the theoretical detection limit of escape frequency was calculated as 1.5 × 10^−12^. This is defined by the ratio between the minimum detectable concentration in induced samples (1.01 × 10° CFU/ml) and the maximum theoretical cell density observed in non-induced controls (6.7 × 10^11^ CFU/ml). All assays were performed in biological triplicates, and results are reported as mean ± standard deviation.

### Long-term viability assay

Long-term viability was assessed using an extended version of the viability assay described earlier. Briefly, cultures were serially passaged for 10 rounds under induced or non-induced conditions. Induction was initiated at an OD_600_ of 1.0 by adding anhydrotetracycline (400 ng/ml), rhamnose (0.5% w/v), and theophylline (5 mM) and maintained at 25°C for 1–2 h depending on the experimental condition. Cultures were diluted 1:1000 at the end of each round to generate seed cultures for the subsequent round. Passage intervals were 12 h per round. CFU determination was performed at each indicated round as described for the viability assay.

The assay was performed across multiple *E. coli* genetic backgrounds (MG1655, W3110, and Nissle 1917) using strains harboring the multi-sgRNA CRISPR BE plasmid (pTet_dCas_CDA_multi). All experiments were carried out in biological triplicates and reported as mean ± s.d.

### Reporter expression interference assay

For reporter expression validation, the eEGM plasmid was co-transformed with an mCherry reporter plasmid carrying ampicillin resistance and either the multi-sgRNA BE plasmid (eEGM) or an empty control vector (pACYCDuet-1). Co-transformants were selected on LB agar containing chloramphenicol (34 μg/ml) and ampicillin (100 μg/ml) and cultured in LB broth supplemented with the same antibiotics for plasmid maintenance. Single colonies were inoculated into LB medium containing appropriate antibiotics and incubated overnight at 37°C with shaking at 250 rpm. The following day, cultures were diluted 1:100 into fresh LB medium and grown until stationary phase to ensure sufficient protein accumulation. An aliquot (200 μl) of each culture was transferred to a 96-well black microplate with a clear bottom. Fluorescence intensity (excitation: 575 nm, emission: 616 nm) and optical density (OD_600_) were measured using a Hidex microplate reader (Hidex, Turku, Finland). Heterologous protein production efficiency was quantified as specific fluorescence, calculated by normalizing the raw fluorescence units (RFU) to the optical density (RFU/OD_600_).

## Results

### Design and selection of start codon-targetable essential genes

**Figure 1.**
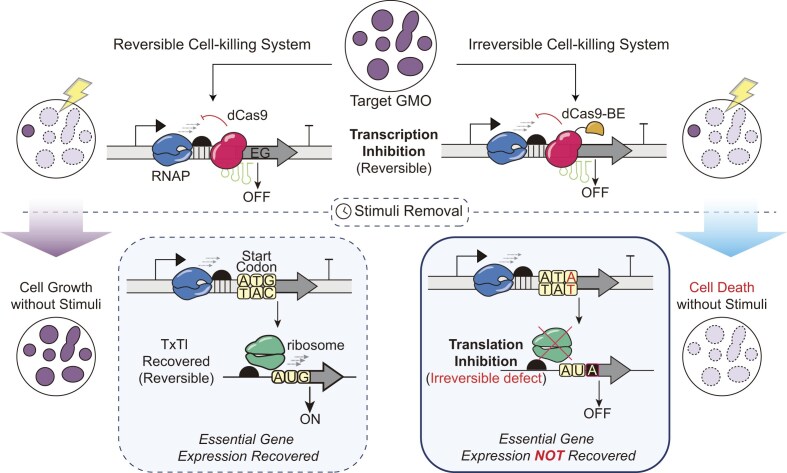
Conceptual framework of irreversible biocontainment using CRISPR-mediated BE. Schematic representation of the reversible and irreversible cell-killing strategies by targeting essential genes (EG). Conventional dCas9-driven cell repression (interference-based Essential Gene targeting, iEG) requires continuous effector occupancy, making it susceptible to growth recovery if the effector expression is removed. In contrast, editing-mediated essential gene targeting (eEG) utilizes a cytidine BE to induce a permanent genetic transition (C-to-T), leading to irreversible translational defect of the target gene. Blue, pink, yellow, and green components denote RNA polymerase, dCas9, cytidine deaminase, and ribosome, respectively. Solid cells indicate viable populations, whereas dotted cells represent non-viable cells.

**Figure 2.**
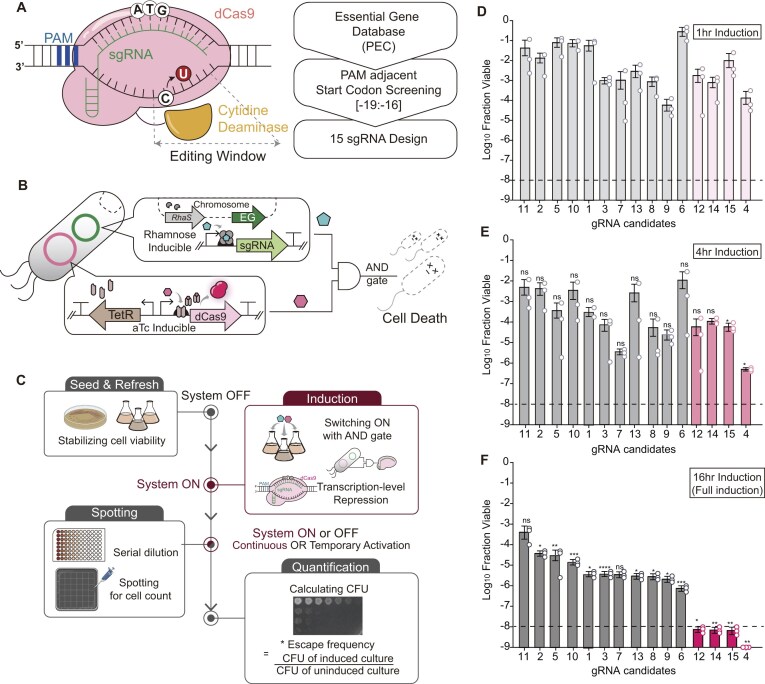
Identification and quantitative evaluation of essential gene-targeting sgRNAs. (**A**) A scheme of the *in silico* screening workflow used to identify start-codon-targetable essential genes in *E. coli*. (**B**) Genetic architecture of the dual-plasmid AND-gate biocontainment circuit in which dCas9 and sgRNA are independently controlled by *Ptet* (aTc) and *Prha* (rhamnose), respectively. (**C**) Experimental workflow for the liquid-to-solid induction and spotting assay used to quantify escape frequencies under varying induction durations. Quantitative evaluation of escape frequencies for the library of 15 candidate sgRNAs at (**D**) 1 h, (**E**) 4 h, and (**F**) 16 h of induction. The *x*-axis indicates individual sgRNAs and the *y*-axis represents fraction of viable cells in log scale. The fraction viable was calculated as the CFU of induced cultures divided by that of uninduced culture. The dashed line represents the NIH guideline criterion (10^−8^). Selected top-performing sgRNAs (sgRNA 4, 12, 14, and 15) targeting distinct essential functions (*holA, ftsB*, and *dfp*) are highlighted. Data are presented as mean ± s.d. from three independent biological replicates (*n* = 3), and the white dots indicate the actual data points. The *P*-value of each strain’s dataset was determined by two-tailed Student’s *t*-test compared to the dataset of 1-h induced conditions, respectively. The asterisk indicates the *P*-value. NS: not significant; *P < .05, **P < .01, ***P < .001, ****P < .0001.

We focused on the start codon (ATG) as a universal control node. Disruption of this site would permanently abolish translation initiation regardless of the downstream sequence, providing a broadly applicable targeting criterion across protein-coding genes [38, 39]. We prioritized cytidine deamination-driven base editing (CBE) over adenine deamination-driven editing because adenine conversion at the +1 position could potentially generate alternative bacterial start codons (e.g. GTG or TTG) [40]. In contrast, conversion of the invariant guanine at the +3 position enables formation of a translation-incompetent triplet (ATG → ATA) without viable alternative initiation [41]. dCas9-mediated transcriptional repression requires sustained effector occupancy due to its reversibility (Fig. 1, left panel). In contrast, CBE-mediated editing enables a permanent translation-null state following a single editing event, thereby decoupling cell killing from continuous effector expression (Fig. 1, right panel). Cytidine BEs in prokaryotes exhibit a defined editing window located ~16–19 nucleotides upstream of a PAM [30]. Therefore, leveraging this positional constraint, we performed a systematic *in silico* screening from the *E. coli* Essential Gene database to identify loci where the invariant guanine of the ATG start codon (specifically the antisense cytosine) fell within the optimal editing window (Fig. 2A). This screening yielded 11 target essential genes and 15 candidate sgRNAs (sgRNA 1–15) spanning diverse essential cellular functions, with predicted on-target activity scores ranging from 36 to 72 [42, 43]. The predicted on-target score reflects the relative likelihood of sgRNA activity (Supplementary Table S1).

To enable quantitative assessment of sgRNA-dependent lethality, we constructed dual-plasmid AND-gate circuits in which dCas9 and sgRNA expression were regulated by two orthogonal inducible promoters, the aTc-inducible *Ptet* and the rhamnose-inducible *Prha*, respectively (Fig. 2B). Both promoters were selected because among the common inducible systems, *Ptet* and *Prha* are known to offer the stringent off-state control required to handle highly toxic gene products [36, 44–46], unlike leaky IPTG-inducible *Ptac* or arabinose-inducible *Para* [47]. This architecture ensured that the kill switch was activated only upon the simultaneous presence of both inducers. Also, by decoupling effector and guide expression, a standardized platform for quantitative comparison across the sgRNA library was established. We further designed an experimental workflow by implementing a two-phase liquid-to-solid induction and spotting assay to precisely control the duration of the induction pulse (Fig. 2C). Escape frequencies were quantified for all 15 sgRNAs under transient (1-h), intermediate (4-h), and full (16-h) induction regimes (Fig. 2D–F).

According to the established assay, cells maintained normal growth in the absence of either inducer, confirming proper tightening of the AND-gate regulation (Supplementary Fig. S1). All sgRNAs exhibited induction-duration-dependent reductions in escape frequency with increasing induction duration, though the magnitude and kinetics varied across constructs. Under transient 1 h induction, escape frequencies ranged from ∼10^−4^ (sgRNA 9) to ∼3 × 10^−1^ (sgRNA 6), indicating limited repression at early time points for most constructs (Fig. 2D). Extending induction to 4 h improved performance across the library, with top-performing sgRNAs, such as sgRNA 4, reaching ∼5 × 10^−7^, whereas others remained at moderate levels (∼10^−2^ to 10^−3^) (Fig. 2E). Under full 16 h induction, escape frequencies converged to substantially lower values, with the best-performing sgRNAs achieving at or below 10^−8^ (Fig. 2F). This delayed repression is likely attributable to the intrinsic kinetics of dCas9-sgRNA complex formation [25] and to target engagement or sufficient accumulation of the dCas9-sgRNA complex to impose CRISPR interference [48, 49].

Notably, distinct kinetic profiles were also observed among sgRNAs. For instance, sgRNA 12 (*ftsB*-1) showed rapid suppression across induction time, whereas sgRNA 6 exhibited a comparatively slower reduction in escape frequency (Fig. 2D–F). These differences likely reflect variation in target-dependent constraints, including local genomic context and the essential burden imposed by repression of distinct cellular pathways [50].

Consistent with this observation, highly effective sgRNAs were preferentially associated with targets involved in critical and non-redundant cellular functions. Based on dCas9-mediated repression efficiency under continuous induction, which serves as a proxy for target accessibility and binding strength, we selected three high-performing sgRNAs for further development: sgRNA 4 (*holA*-2; 3.0 × 10^−10^), sgRNA 12 (*ftsB*-1; 7.7 × 10^−9^), and sgRNA 15 (*dfp*-2; 6.7 × 10^−9^). Although sgRNA 14 (*dfp*-1) exhibited a comparable escape frequency (7.0 × 10^−9^), sgRNA 12 (*ftsB-*1) was prioritized to avoid redundancy within the same locus. These selected targets span functionally distinct essential processes, including DNA replication (*holA*), cell division (*ftsB*), and metabolic cofactor biosynthesis (*dfp*), each of which imposes strong fitness constraints upon repression [51]. This pathway-level diversity was intentionally incorporated to maximize the cumulative essential burden and thereby reduce the likelihood of escape. Accordingly, a triad of sgRNAs targeting these orthogonal essential pathways was implemented to establish a robust and irreversible CRISPR–dCas9-driven biocontainment system.

### Enhancement of biocontainment through base editing

**Figure 3.**
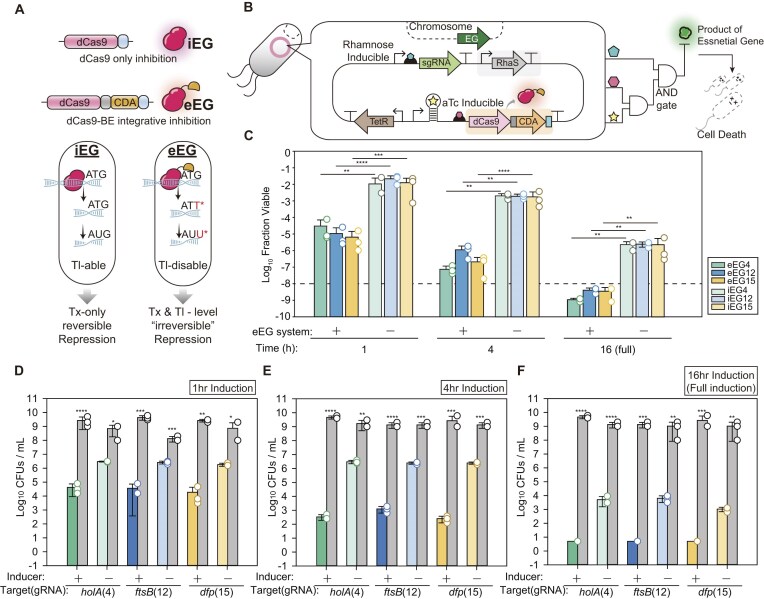
Implementation of irreversible biocontainment via BE-mediated essential gene targeting. (**A**) Schematic comparison between interference-based essential gene targeting (iEG) system and editing-based essential gene targeting (eEG) system targeting the start codon of essential gene. The fused *PmCDA* catalyzes a permanent C-to-T transition at the start codon of the target essential gene, leading to irreversible translational inactivation. Binding-only system on the target site enables reversible cell recovery, while editing the start codon by eEG system disables the translation irreversibly. (**B**) Genetic architecture of the single-plasmid construct which consists of editing-based essential gene targeting (eEG) circuit. The BE and sgRNA are co-expressed within a single construct under inducible systems, respectively. (**C**) Comparison of escape frequencies between the transcriptional interference (iEG) and editing-based (eEG) systems for selected targets (4, 12, and 15 refers to system targeting *holA, ftsB*, and *dfp*, respectively). Cell repression ratio was measured following transient induction time (1, 4, and 16 h). Colors indicate different sgRNA targets (green, blue, and yellow), while shade intensity distinguishes the two systems (light, iEG; dark, eEG). The dashed line indicates the NIH guideline criterion (10^−8^). (**D**–**F**) Assessment of off-state stability and induction-dependent cell viabilities. Population densities (CFU/ml) of the eEG system were quantified for (D) sgRNA 4, (E) sgRNA 12, and (F) sgRNA 15 under 1, 4, and 16 h induction conditions. Grey bars represent non-induced populations, showing stable cell viability (∼10^9^ CFU/ml), while colored bars indicate escape populations post-induction. Data are presented as mean ± s.d. from three independent biological replicates (*n* = 3), and the white dots indicate the actual data points. The asterisk indicates the *P*-value determined by the two-tailed Student’s *t*-test. NS: not significant; *P < .05, **P < .01, ***P < .001, ****P < .0001.

While dCas9-mediated transcriptional interference (iEG) provides a versatile platform for gene repression [25, 52], its inherent reversibility necessitates sustained effector presence [9, 53]. To overcome this limitation, we designed an editing-based essential gene targeting (eEG) architecture utilizing dCas9 fused to a cytidine deaminase (*PmCDA1*) to catalyze a permanent genetic transition at the translation initiation site (Fig. 3A). We also employed a *PmCDA1* variant featuring a C-terminal LVA degradation tag (AANDENYALVA) to mitigate the inherent cellular toxicity and unintended genomic alterations associated with basal CDA activity [30].

First, we validated the mechanistic activity of this eEG-mediated disruption targeting a genome-integrated *mCherry* reporter system. Using the previous construct, a dual-plasmid system, we observed premature *mCherry* repression even in the absence of inducers, leading to the rapid emergence of non-functional mutants (Supplementary Fig. S2). This result suggests that the high regulatory threshold in this configuration should be considered given the irreversible nature of BE. Thus, we optimized the circuit by integrating the effector and sgRNA into a single low-copy vector and incorporating a theophylline-responsive riboswitch downstream of the *Ptet* promoter [54] (Fig. 3B). In this architecture, system activation is governed by a multi-layered regulatory scheme in which each component is independently gated at distinct regulatory levels. Expression of the dCas9–BE is controlled at both the transcriptional level via an aTc-inducible promoter and the translational level via a theophylline-responsive riboswitch, while sgRNA expression is independently driven by a rhamnose-inducible promoter. Together, these orthogonal controls implement an effective AND-gate logic, such that functional editing activity is achieved only when all inputs are simultaneously present, thereby minimizing unintended basal activity. This triple-layered control effectively sequestered the editing machinery, as evidenced by the stable *mCherry* expression in the non-induced state and the irreversible phenotypic loss observed following a 16-h induction pulse (Supplementary Fig. S3A–D). Subsequent Sanger sequencing of the sorted low-fluorescence population confirmed precise C-to-T transitions within the base-editing window, specifically disrupting the start codon (ATG to ATA). While some mutations were observed in the adjacent 5′ UTR region (Supplementary Fig. S3E), the predominant phenotypic loss was attributed to the robust inactivation of the initiation codon.

Building upon the validation, we applied the eEG architecture to target endogenous essential genes with three previously selected top-performing candidates, sgRNA 4, 12, and 15 (Fig. 3C). The iEG system exhibited a marked decrease in repression efficiency compared to the initial screening results (Fig. 2D–F), with escape frequencies remaining relatively high even under full 16-h induction (e.g. 2.43 × 10^−6^ for iEG4). We attribute the reduced repression efficiency of the iEG system relative to the initial screening experiments to decreased effective dCas9 activity, likely resulting from the transition to a low-copy backbone and the implementation of a multi-layered regulatory architecture, including additional translational regulations imposed by the riboswitch. These together likely lower intracellular dCas9 below the binding-saturation regime. Meanwhile, the eEG system maintained robust performance, with escape frequencies reaching as low as 1.11 × 10^−9^ (eEG4) under the same 16-h induction regime, representing a ∼2000-fold increase in containment stringency compared to iEG.

While irreversible transitions of eEG offer superior efficacy over the transient occupancy of dCas9, the high catalytic turnover of deaminases has the possibility of cumulatively imposing a fitness cost under the OFF state [55, 56]. To determine whether our multi-layered control module effectively sequesters the editing machinery in the OFF state, we monitored the viability (CFU/ml) of non-induced populations following various induction pulses (Fig. 3D–F). Notably, across all tested sgRNAs, no significant reduction in cell viability was observed in the absence of inducers (*P* > .05), and population densities remained consistently stable at ∼10^9^ CFU/ml. This observation confirms that the cumulative genetic burden of the eEG system remains below the threshold for homeostatic impairment.

### Multiplexed base editing for accelerated containment and improved robustness

**Figure 4.**
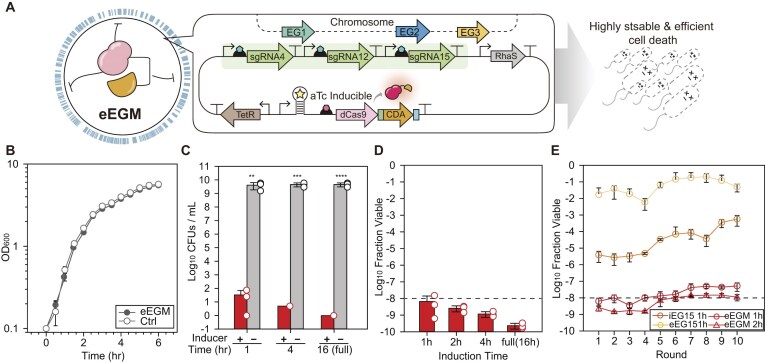
Multiplexed BE for enhanced biocontainment stringency. (**A**) Schematic representation of the multiplexed eEG (eEGM) system. The system simultaneously targets three independent essential loci (EG1, EG2, and EG3) to achieve synergistic cell killing and prevent mutational escape. (**B**) Growth kinetics (OD_600_, log scale) of control and eEGM-bearing strains under non-induced conditions. Ctrl refers to the MG1655 strain carrying a non-circuit control plasmid (pACYC) containing the same p15A origin and chloramphenicol resistance marker. (**C**) Population density (CFU/ml) of the eEGM system to assess the off-state stability and escape frequency under full induction. Grey bars represent non-induced populations, showing stable cell viability (∼10^9^ CFU/ml), while colored bars indicate escape populations post-induction. (**D**) Killing kinetics among diverse induction durations in multiplexed eEGM system. (**E**) Long-term evolutionary stability assay over 10 sequential passages. Single-target systems (orange circle, 1-h induced iEG15; yellow circle, 1-h induced eEG15) and the multiplexed eEGM system (red circle, 1-h induced eEGM; red triangle, 2-h induced eEGM) were compared. Data are presented as mean ± s.d. from three independent biological replicates (*n* = 3), and the white dots indicate the actual data points. The dashed line indicates the NIH guideline criterion (10^−8^). The asterisk indicates the *P*-value determined by the two-tailed Student’s *t*-test. NS: not significant; *P < .05, **P < .01, ***P < .001, ****P < .0001.

Although eEG exhibited enhanced repression compared to iEG, single-locus targeting still required a full 16-h induction to meet the safety threshold. We hypothesized that multiplexing the independent essential targets may lower the absolute escape frequency by significantly accelerating the killing kinetics. Building upon this, we engineered a multiplexed eEG (eEGM) system simultaneously targeting three independent essential loci (EG1, EG2, and EG3) (Fig. 4A). Despite the increased genetic load of expressing multiple sgRNAs, the eEGM-bearing strains exhibited growth kinetics (μ_max_, 1.65 h^−1^; doubling time, 0.42 h) comparable to the control group (μ_max_, 1.47 h^−1^; doubling time, 0.47 h) in the absence of inducers (*P* > .05), maintaining their cellular fidelity (Fig. 4B). The stringent sequestration of the editing machinery was further confirmed by stable CFU counts over 16 h without induction, where population densities remained consistently at ∼10^9^ CFU/ml (Fig. 4C).

Acceleration of killing kinetics was significantly observed in the eEGM architecture compared to single-locus targeting. While the single-gene targeting eEG system failed to meet the NIH guideline criterion (10^−8^) under short-term induction, the eEGM system surpassed the standard within 1-h induction (Fig. 4D). This rapid transition is attributed to the synergistic effect of simultaneous C-to-T transitions across multiple loci, which significantly lowers the probability of stochastic survival. As expected, the escape frequency of eEGM dropped to 1.11 × 10^−10^ under full induction (Fig. 4C), effectively reaching the limit of detection for our assay.

To further evaluate the long-term evolutionary stability of the eEGM safeguard, we performed a sequential passage assay over 10 rounds, comparing it against interference-based (iEG15) and single-locus editing (eEG15) systems (Fig. 4E). While both the iEG15 and eEG15 system targeting single locus showed rapid failure within five rounds due to the rapid emergence of escape mutants, the eEGM system maintained an escape frequency below 10^−8^ throughout the entire experiment, even with short 1- or 2-h induction pulses. Taken together, the eEGM system effectively repressed the cellular population with its stable off-state control and preserved stability across generations.

### Cross-strain portability and functional orthogonality of the eEGM system

**Figure 5.**
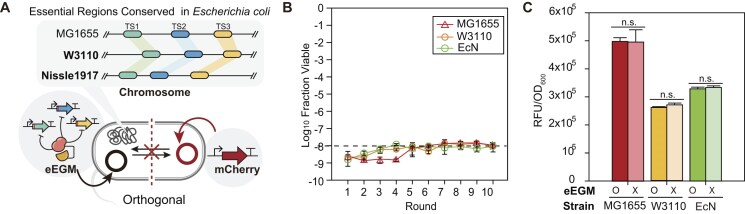
Cross-strain portability and functional orthogonality of eEGM system. (**A**) Schematic representation of the eEGM module design for cross-strain application and orthogonality testing. Three essential target sites (TS1, *holA*; TS2, *ftsB*; TS3, *dfp*) are highly conserved among *E. coli* MG1655, W3110, and Nissle 1917 (EcN). An independent *mCherry* reporter system was co-expressed to monitor potential metabolic interference under off-state (pre-induction) conditions. (**B**) Escape frequencies of *E. coli* MG1655 (red, triangle), W3110 (orange, circle), and EcN (green, circle) over 10 serial passages. The dashed line indicates the NIH guideline criterion (10^−8^). (**C**) Metabolic orthogonality evaluated by quantifying fluorescence of the constitutively expressed reporter *mCherry*. Specific fluorescence (RFU/OD_600_) between strains with (eEGM+) and without (eEGM−) the containment module were compared. The negligible difference in mCherry production across all three strains refers to the functional transparency of the system. The error bar represents the mean ± standard deviation from the biologically independent cell cultures (*n* = 3), and the white dots indicate the actual data points. Data are presented as mean ± s.d. from three independent biological replicates (*n* = 3). The dashed line indicates the NIH guideline criterion (10^−8^). The asterisk indicates the *P*-value determined by the two-tailed Student’s *t*-test. NS: not significant; *P < .05, **P < .01, ***P < .001, ****P < .0001.

Synthetic constructs often exhibit inconsistent performance due to strain-specific genomic polymorphisms or restriction-modification barriers [57, 58]. Likewise, the implementation of a universal biocontainment platform requires functional consistency across diverse genetic backgrounds. We therefore examined the applicability of our design across three representative strains: MG1655 (laboratory model) [59], W3110 (industrial chassis) [60], and Nissle 1917 (EcN, therapeutic vehicle) [61]. Sequence alignment revealed that the target sequences for the three selected sgRNAs (sgRNA 4, 12, and 15) and their corresponding essential gene loci are 100% conserved across MG1655, W3110, and EcN (Fig. 5A and Supplementary Fig. S4). Upon 2-h short-term induction, all tested strains consistently maintained escape frequencies within a narrow range (10^−8^ to 10^−10^) below the NIH guideline criterion (10^−8^) throughout 10 sequential rounds (Fig. 5B). Considering the fact that the strains represent distinct physiological niches [62–64], these observations suggest that the efficacy of the eEGM system is largely independent of host-specific physiological differences among the tested strains.

Furthermore, we investigated the functional orthogonality of the eEGM system to ensure it remains “silent” during the production phase. As anticipated from prior characterizations of *E. coli* lineage-specific traits [65], we observed significant metabolic heterogeneity among the three strains. The baseline mCherry intensity varied substantially, with MG1655 exhibiting nearly twofold higher expression than W3110 and EcN (4.99 × 10^5^ versus 2.61 × 10^5^ and 3.30 × 10^5^ RFU/OD_600_, respectively; *P* < .05), reflecting the divergent metabolic flux and ribosomal capacities inherent to these lineages [65, 66] (Fig. 5C). Despite these diverse metabolic profiles, the inactive eEGM machinery imposed no significant burden on heterologous protein synthesis. The variance in fluorescence between eEGM-bearing and eEGM-free cells was negligible across all strains (3.7% in W3110, 1.2% in EcN, and < 0.5% in MG1655, *P* > .05) (Fig. 5C). This lack of resource competition indicates that the dCas9–CDA complex is successfully sequestered in the OFF-state, ensuring that the safeguard does not compromise the host’s engineered functions. This decoupling of containment efficacy from host-specific translational capacity is noteworthy, ensuring that even in strains with lower protein synthesis rates, the eEGM system maintains comparable containment efficiency. These results establish the eEGM system as a highly portable and metabolically transparent platform, effective regardless of the host’s unique physiological or biosynthetic constraints.

## Discussion

The eEGM platform established in this study addresses a fundamental bottleneck in synthetic biology: the trade-off between the tightness of a biocontainment switch and its physiological burden on the host. While previous nuclease-based strategies have focused on inducing catastrophic DNA double-strand breaks, these systems often suffer from leaky basal expression, leading to unintended genomic instability or strong selective pressure for mutational bypass [18, 67]. Our findings demonstrate that utilizing a CBE to transition the start codon (ATG) of essential genes into non-functional triplets provides an effective alternative by creating an irreversible translational blockade. Unlike dCas9-based interference (CRISPRi), which requires the continuous presence of an inducer to maintain repression [25], the eEGM module achieves a terminal state with only a transient pulse of induction. This kinetic profile is particularly advantageous for environmental or clinical scenarios where sustained inducer concentration cannot be guaranteed. Notably, this transient activation scheme also has implications for the potential risks associated with cytidine BEs. BEs have been reported to induce a general risk of genome-wide off-target mutations [68, 69], yet the functional consequences of these effects are likely to be mitigated in this system. This can be understood in the context of the limited duration of editing activity and the programmed transition to a non-proliferative state that ultimately leads to cell elimination, thereby restricting the propagation and enrichment of off-target mutations.

A key mechanistic insight from our study is the substantial enhancement of containment stringency through multiplexed targeting of non-redundant essential pathways. By simultaneously targeting multiple loci, we bypassed the stochastic nature of single-locus repair or alternative translation initiation, which has been a primary cause of escaper emergence in earlier designs [11]. Importantly, achieving escape frequencies below 10^−8^ was compatible with metabolic orthogonality, consistent with minimal OFF-state burden of the sequestered dCas9–CDA complex. In addition, this circuit architecture reconciles long-term genetic stability with minimal fitness cost, establishing a robust framework for irreversible biocontainment without nuclease-mediated toxicity. Our evaluation with the *mCherry* reporter system also confirms that the dCas9–CDA fusion complex, when sequestered by the theophylline-responsive riboswitch, imposes negligible competition for the host’s transcriptional or translational machinery [70, 71]. This functional orthogonality is a prerequisite for complex metabolic engineering, where any deviation in host fitness can lead to unpredictable yields in heterologous protein or metabolite production [72–74].

While the current eEGM module targets highly conserved essential genes in *E. coli*, the programmable nature of sgRNA design allows for rapid adaptation to other species in principle, by redesigning the sgRNA targeting sequence, highlighting the inherent modularity of our base-editing platform. The complete conservation of the target protospacer and PAM sequences suggests that the eEGM system can be deployed as a readily transferable safeguard across various *E. coli* lineages without the need for strain-specific modifications. Although we demonstrate robust portability across *E. coli* lineages, extending this strategy to other species will require re-optimization of regulatory parts and editing parameters to account for differences in DNA repair pathways and genomic context [75, 76]. Practical constraints such as PAM availability [77, 78], base-editing window limitations [79, 80], variability in essential gene conservation, and host-dependent editing efficiency should also be systematically addressed in future studies. In this regard, while the escape frequencies achieved at the empirically selected concentrations meet NIH standards, systematic fine-tuning could further broaden the operational window by optimizing the trade-off between editing efficacy and metabolic burden. Furthermore, since we identified only three efficient target sites targeting start codons, integrating PAM-flexible Cas variants or engineered CDAs could expand the targetable repertoire to include essential regulatory motifs or active-site residues [78, 81].

The rapid expansion of the CRISPR-Cas9 toolkit across diverse microbial chassis also provides a strong foundation for the system’s scalability. High-efficiency genome engineering has already been established in various industrial and therapeutic models, including *Bacillus subtilis* [26], *Pseudomonas putida* [82], *Lactobacillus* species [83], and *Corynebacterium glutamicum* [84]. These existing functional validations suggest that the eEGM module can be adapted as a versatile safeguard by swapping current promoters and sgRNA scaffolds with established broad-host-range or genus-specific genetic parts. This cross-species compatibility will be pivotal for the secure deployment of genetically GEMs in complex, multi-species environments. Future studies should also evaluate the stability of these genomic edits under fluctuating selective pressures in semi-closed ecosystems to ensure long-term evolutionary permanence. This framework provides a practical foundation for safer deployment of next-generation live biotherapeutics and industrial GEMs.

Despite the need for further validation in complex ecological backgrounds, this study expands the operational space of programmable biocontainment by establishing base-editing as a programmable, nuclease-independent effector that enables durable genetic containment through transient activation. Our results demonstrate that eEGM provides a versatile safeguard by decoupling robust biocontainment from continuous induction and host-specific chromosomal constraints. This portable system remains fully compatible with complex metabolic engineering strategies across various microbial chassis, establishing a modular and operationally efficient CRISPR effector for stringent biocontainment through brief induction.

## Supplementary Material

gkag422_Supplemental_File

## Data Availability

All data supporting the findings of this study are available within the paper and its online supplementary material.

## Code availability

Custom Python scripts used for essential gene screening and PAM-site identification are publicly available at GitHub repository (https://github.com/swon0912/EG-start-target-sgRNA-screening/releases/tag/v1.0) and archived at Zenodo (DOI 10.5281/zenodo.18784974).
