# Supplementary material for: Multiplexed CRISPR base editing enables pulse-activated irreversible biocontainment of engineered bacteria
Source: Nucleic Acids Res. 2026 May 4;54(8):gkag422. doi: 10.1093/nar/gkag422 (PMC13136896; doi:10.1093/nar/gkag422)
Supplement: gkag422_Supplemental_File [file gkag422_supplemental_file.pdf]

Supplementary Information

Multiplexed CRISPR base editing enables pulse-activated irreversible biocontainment of engineered bacteria

Sung Won Cho[†1], TaeHyun Kim[†1], Jina Yang[2], Gibyuk Byun[1], and Sang Woo Seo[1,2,4,5,6,*]

[1]Department of Chemical and Biological Engineering, Seoul National University, 1 Gwanak-ro, Gwanak-Gu, Seoul 08826, Republic of Korea

[2]Department of Chemical Engineering, Jeju National University, 102, Jejudaehak-ro, Jeju-si, Jeju-do 63243, South Korea

[3]Interdisciplinary Program in Bioengineering, Seoul National University, 1 Gwanak-ro, Gwanak-gu, Seoul 08826, South Korea

[4]Institute of Chemical Processes, [5]Bio-MAX Institute, and [6]Institute of Bio Engineering, Seoul National University, 1 Gwanak-ro, Gwanak-gu, Seoul 08826, South Korea

[†]These authors contributed equally to this work.
[*]To whom correspondence.

(S.W.S) School of Chemical and Biological Engineering, Seoul National University, 1 Gwanak-ro, Gwanak-Gu, Seoul, 08826, South Korea.

Tel.: +82 2 880 2274; Fax: +82 2 888 7295; Email: swseo@snu.ac.kr

Supplementary Tables

Supplementary Table S1. Start codon-targetable sgRNAs screened in this study

GeneSyb	Direction	Upper (5'->3')	Description	gRNA Label	gRNA	[a]Predicted On-target activity score		[d]Centroid ($\Delta G_{centroid}$, kcal/mol)	Label
						[b]Doench 2016	[c]Moreno-Mateos 2015		
ispH	+	TGGAAATCGATCCGGCACTGGAGGCGTAAC	1-hydroxy-2-methyl-2-(E)-butenyl 4-diphosphate	ispH	GCATGTTACGCCTCCAGTGC	53	46	-15.4	01_gRNA-R_ispH
thiL	+	TTTTCTTTTTTACCTGCTGAGGCATAACGT	thiamine monophosphate kinase	thiL	GCCATACGTTATGCCTCAGC	55	48	-20.9	02_gRNA-R_thiL
holA	-	CTGCACGCGTCTCCACCACGCTGGGTAACTG	DNA polymerase III subunit delta	holA-1	TCATCAGTTACCCAGCGTGG	66	51	-15.4	03_gRNA-R_holA-1
				holA-2	GAATCATCAGTTACCCAGCG	72	51	-12.1	04_gRNA-R_holA-2
lolD	+	CCGCCACTCAACCCGCTGAGGCTTTACGTT	lipoprotein release complex-ATP binding	lolD-1	TCATAACGTAAAGCCTCAGC	54	44	-25.4	05_gRNA-R_lolD-1
				lolD-2	TTCATAACGTAAAGCCTCAG	63	46	-26.9	06_gRNA-R_lolD-2
lolE	+	GGCGGAACTGAGCCTGATGGGGGCGGAGTA	lipoprotein release complex-inner membrane subunit	lolE	GCCATTACTCCGCCCCCATC	40	33	-17.8	07_gRNA-R_lolE
folE	-	GGAGGCGTCACACCTGCAGGAGAAATCATAA	GTP cyclohydrolase I	folE	GCATTTATGATTTCTCCTGC	45	12	-20.1	08_gRNA-R_folE
ffh	-	GGCCAACCGTTTCCACCCCAGGCGAGAGACA	signal recognition particle protein component	ffh	ACATTGTCTCTCGCCTGGGG	54	55	-16.9	09_gRNA-R_ffh
ispF	-	TCACCCGAACCATCCATCAGGAGAATACATA	2-C-methyl-D-erythritol 2,4-cyclodiphosphate	ispF-1	TTATGTATTCTCCTGATGGA	54	32	-17	10_gRNA-R_ispF-1
				ispF-2	CGCATTATGTATTCTCCTGA	49	15	-19.9	11_gRNA-R_ispF-2
ftsB	-	GGGATGATGATGCCGTTTTTCAGGGGGCAGG	Cell division protein	ftsB	CCATCCTGCCCCCTGAAAAA	36	39	-17.5	12_gRNA-F_ftsB
rpmA	-	TCAGCGCCTAAGACCTGAGGAGAGATTTCAA	50S ribosomal subunit protein L27	rpmA	GCCATTTGAAATCTCTCCTC	48	39	-16.3	13_gRNA-F_rpmA

dfp	+	TTCTGGTGTCA**CCC**TACAGGAAAAATCATC	fused 4'-phosphopantothenoylcysteine decarboxylase and phosphopantothenoylcysteine synthetase	dfp-1	TCATGATGATTTTTCCTGTA	45	56	-21.3	14_gRNA-F_dfp-1
				dfp-2	CTCATGATGATTTTTCCTGT	50	20	-23.1	15_gRNA-F_dfp-2

[a] Predicted On-target activity score: Scores indicate the predicted relative activity of each sgRNA according to the respective models.

[b] Predicted on-target activity score calculated by Doench et al. (1)

[c] Predicted on-target activity score calculated by Moreno-Mateos et al. (2)

[d] Centroid ($\Delta G_{centroid}$, kcal/mol): The free energy of the centroid secondary structure, which represents the conformation with the minimum total distance to all other structures in the thermodynamic ensemble. This value was used to assess the structural accessibility of the guide sequence.

Supplementary Table S2. Strains and plasmids used in this study

Name	Relevant characteristics	Source
	**Strains**	
Escherichia coli K-12 MG1655	F^- wild type	Laboratory stock (Herring et al, 2006)
MG1655-mch	*Escherichia coli* K-12 MG1655::P_{OXB20}-mCherry-$T_{L3S2P56}$	This study
Escherichia coli W3110	F^-, λ^-, rph-1, *rrnD-rrnE*, *ilvG*$^-$	Laboratory stock (Hayashi et al, 2006)
Escherichia coli Nissle 1917	Serotype O6:K5:H1, *mutS*$^+$, *fliC*$^+$, pMUT1, pMUT2	Prof. Matthew Chang, National University of Singapore (Hwang et al, 2017)
Mach1-T1^R	F^- φ80(*lac*Z)ΔM15 Δ*lac*X74 *hsd*R($r_K^- m_K^+$) Δ*rec*A1398 *end*A1 *ton*A	Invitrogen
	**Plasmids**	
pdCas9	pdCas9_Ptet_dCas9, CmR, p15A ori	Addgene #44249
pCas_CDF	pCas-Ptrc-sgRNA pMB1::sgRNA-pCDF	https://doi.org/10.1016/j.celrep.2021.109589
pCDF_MgG1	pCDF-Duet backbone, J23119 promoter, sgRNA targeting chromosomal intergene	This study
pCDF_sgRNA_#	pCDF-Duet backbone, J23119 promoter, sgRNA # (1 to 15) respectively	This study
pTet_dCas_CDA_sgRNA_4	pACYC_pTet_dCas_BE_pRha_sgRNA4	This study
pTet_dCas_CDA_sgRNA_12	pACYC_pTet_dCas_BE_pRha_sgRNA12	This study
pTet_dCas_CDA_sgRNA_15	pACYC_pTet_dCas_BE_pRha_sgRNA15	This study
pTet_dCas_CDA_sgRNA_mcherry	pACYC_pTet_dCas_BE_pRha_sgRNAmcherry	This study
pTet_dCas_CDA_sgRNA_multi	pACYC_pTet_dCas_BE_pRha_sgRNA4_pRha_sgRNA12_pRha_shRNA15	This study
pTet_dCas_LVA_sgRNA_4	pACYC_pTet_dCas_pRha_sgRNA4	This study
pTet_dCas_LVA_sgRNA_12	pACYC_pTet_dCas_pRha_sgRNA12	This study
pTet_dCas_LVA_sgRNA_15	pACYC_pTet_dCas_pRha_sgRNA15	This study
pTet_dCas_LVA_sgRNA_mcherry	pACYC_pTet_dCas_pRha_sgRNAmcherry	This study
pTet_dCas_LVA_sgRNA_mulit	pACYC_pTet_dCas_pRha_sgRNAmcherry	This study
pTet_switch_dCas_CDA_sgRNA_4	pACYC_pTet_switch_dCas_BE_pRha_sgRNA4	This study
pTet_switch_dCas_CDA_sgRNA_12	pACYC_pTet_switch_dCas_BE_pRha_sgRNA12	This study
pTet_switch_dCas_CDA_sgRNA_15	pACYC_pTet_switcht_dCas_BE_pRha_sgRNA15	This study
pTet_switch_dCas_CDA_sgRNA_mcherry	pACYC_pTet_switch_dCas_BE_pRha_sgRNAmCherry	This study

pTet_switch_dCas_CDA_sgRNA_mulit	pACYC_pTet_dCas_BE_pRha_sgRNA4_pRha_sgRNA12_pRha_sgRNA15	This study
pTet_switch_dCas_LVA_sgRNA_4	pACYC_pTet_switch_dCas_pRha_sgRNA4	This study
pTet_switch_dCas_LVA_sgRNA_12	pACYC_pTet_switch_dCas_pRha_sgRNA12	This study
pTet_switch_dCas_LVA_sgRNA_15	pACYC_pTet_switch_dCas_pRha_sgRNA15	This study
pTet_switch_dCas_LVA_sgRNA_mcherry	pACYC_pTet_switch_dCas_pRha_sgRNAmCherry	This study
pTet_switch_dCas_LVA_sgRNA_mulit	pACYC_pTet_switch_dCas_pRha_sgRNA4_pRha_sgRNA12_pRha_sgRNA15	This study
RhaS_pTet_switch_dCas_CDA_sgRNA_multi	Rhas_pACYC_pTet_dCas_BE_pRha_sgRNA4_pRha_sgRNA12_pRha_sgRNA15	This study

Supplementary Table S3. Oligonucleotides used in this study

Name	Sequence (5'-3')
dCas9_BamHI_F	tcgtaaaaaagactgggatcc
dCas_end_R	gtcacctcctagctgact
BE_overhang_Cas_F	tgagtcagctaggaggtgacggtggaggaggttctggag
BE_end_R	ttatgcaaccagtcctagcatc
cas_frag_over_BE_F	ctaggactggttgcataactcgagtaaggatctccagg
cas_frag_R	tataaacgcagaaaggccc
over_sgRNA_single_F	ggcctttctgcgtttatatgttatatcccggccgttaac
sgRNA_over_Avr_R	gaagcggaatatatccctagggccgcttatgtctattgct
sgRNA_UNS4_over_F	ggcctttctgcgtttatagtaagacaacacgcaaagtc
Cas_multi_re_homology_F	gggcctttctgcgtttatactcctgccagcaatagtaag
dCasWT_aatafl_F	cgtctcattttcgccagatatc
dCas_switch_R	gctgccaagggcatcaagacgatgctggtatcaccggtaccttctctatcactgatagggagtg
dCas_switch_F	gtcttgatgcccttggcagcaccctgctaaggaggcaacaagatggataagaaatactcaataggc
dCasWT_Sph_R	gataaaagtcttcttgtcttctc
1st_tet_utr_RhaS_F	tcagtgatagagaaaacctcaatctcttaaggcttcgtcaatgaccgtattacatagtgtgg
2nd_tet_utr_rhaS_F	tgttactagtgttgacactctatcgttgatagagttattttaccactccctatcagtgatagagaaaacctcaatctc
RhaS_SpeI_R	ttttactagtttattgcagaaagccatccc
MgG1_check_F	catcggaatatggcgca
MgG1_check_R	ggctgcccgaaacacc
MGg1_Hom_F(mCherry homologous editing template)	cgctatcgctgagatctgcctttgccggatgcgatgctgacgcatcttatggttttcccagtcacgac
MGg1_Hom_R	ggtggtgagtaagtagaaatcggcggccgcctgcggttgattgccggatgcaggaaacagctatgac
gRNA1_F	tagcgcatgttacgcctccagtgc
gRNA1_R	aaacgcactggaggcgtaacatgc
gRNA2_F	tagcgccatacgttatgcctcagc
gRNA2_R	aaacgctgaggcataacgtatggc
gRNA3_F	tagctcatcagttacccagcgtgg
gRNA3_R	aaacccacgctgggtaactgatga
gRNA4_F	tagcgaatcatcagttacccagcg
gRNA4_R	aaaccgctgggtaactgatgattc
gRNA5_F	tagctcataacgtaaagcctcagc
gRNA5_R	aaacgctgaggctttacgttatga
gRNA6_F	tagcttcataacgtaaagcctcag
gRNA6_R	aaacctgaggctttacgttatgaa
gRNA7_F	tagcgccattactccgcccccatc
gRNA7_R	aaacgatgggggcggagtaatggc

gRNA8_F	tagcgcatttatgatttctcctgc
gRNA8_R	aaacgcaggagaaatcataaatgc
gRNA9_F	tagcacattgtctctcgcctgggg
gRNA9_R	aaaccccccaggcgagagacaatgt
gRNA10_F	tagcttatgtattctcctgatgga
gRNA10_R	aaactccatcaggagaatacataa
gRNA11_F	tagccgcattatgtattctcctga
gRNA11_R	aaactcaggagaatacataatgcg
gRNA12_F	tagcccatcctgccccctgaaaaa
gRNA12_R	aaactttttcagggggcaggatgg
gRNA13_F	tagcgccatttgaaatctctcctc
gRNA13_R	aaacgaggagagatttcaaatggc
gRNA14_F	tagctcatgatgatttttcctgta
gRNA14_R	aaactacaggaaaaatcatcatga
gRNA15_F	tagcctcatgatgatttttcctgt
gRNA15_R	aaacacaggaaaaatcatcatgag
gRNA_mcherry_F	tagccatgttatcctcctcgccct
gRNA_mcherry_R	aaacagggcgaggaggataacatg
gRNA_MgG1_F	tagcaataaattgcagcgttctgt
gRNA_MgG1_R	aaacacagaacgctgcaatttatt

Supplementary Figures

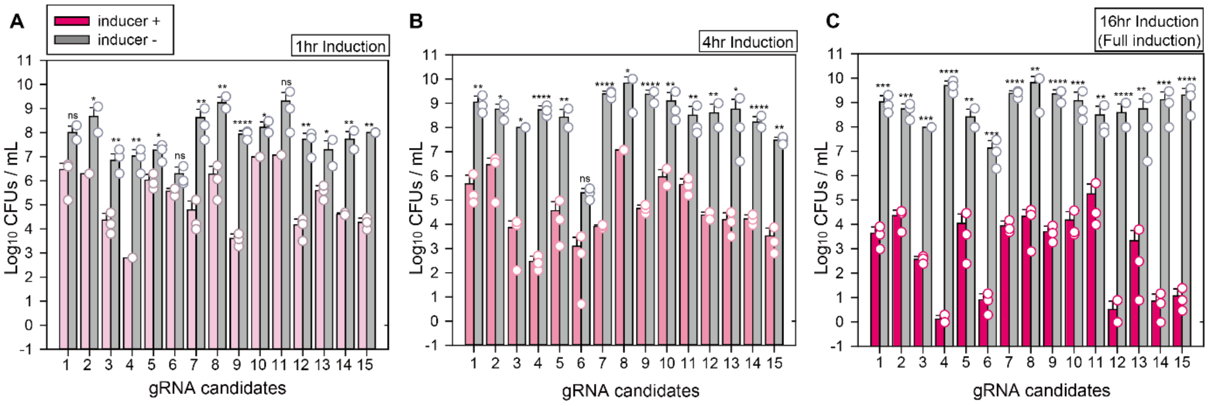

Fig. S1 Time-resolved cell density profiling of sgRNA candidates targeting essential genes. Colony-forming unit (CFU) counts obtained from 15 sgRNA candidates targeting essential genes under three induction regimes: (a) 1h, (b) 4h, and (c) 16h full induction. Grey bars represent non-induced conditions, and colored bars indicate induced conditions. Bars represent mean values from three independent biological replicates (n = 3), and white dots indicate individual data points. These raw survival counts were subsequently used to calculate escape frequencies shown in Fig. 3.

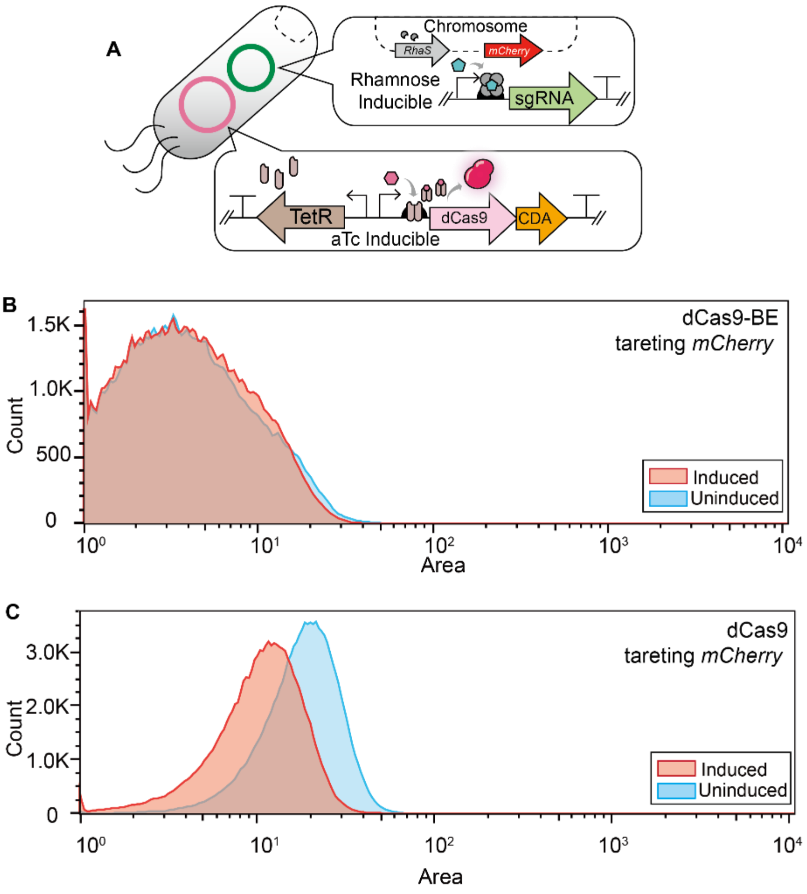

Fig. S2 Basal repression observed in the dual-plasmid base-editing configuration. (a) Representation of the dual-plasmid system in which dCas9-cytidine deaminase (dCas9-BE) and the sgRNA targeting the 5’ UTR of *mCherry*. Circuits were co-expressed from separate inducible constructs. (b) Flow cytometry analysis of cells harboring dCas9-BE and expressing *mCherry* fluorescence. (c) Flow cytometry analysis of cells harboring dCas and expressing *mCherry* fluorescence. Induced (red) and uninduced (blue) populations are demonstrated. Cells were harvested after 4 hours of induction, followed by 16 hours of recovery in media without inducers. Approximately 1×10^5 events were acquired for the analysis.

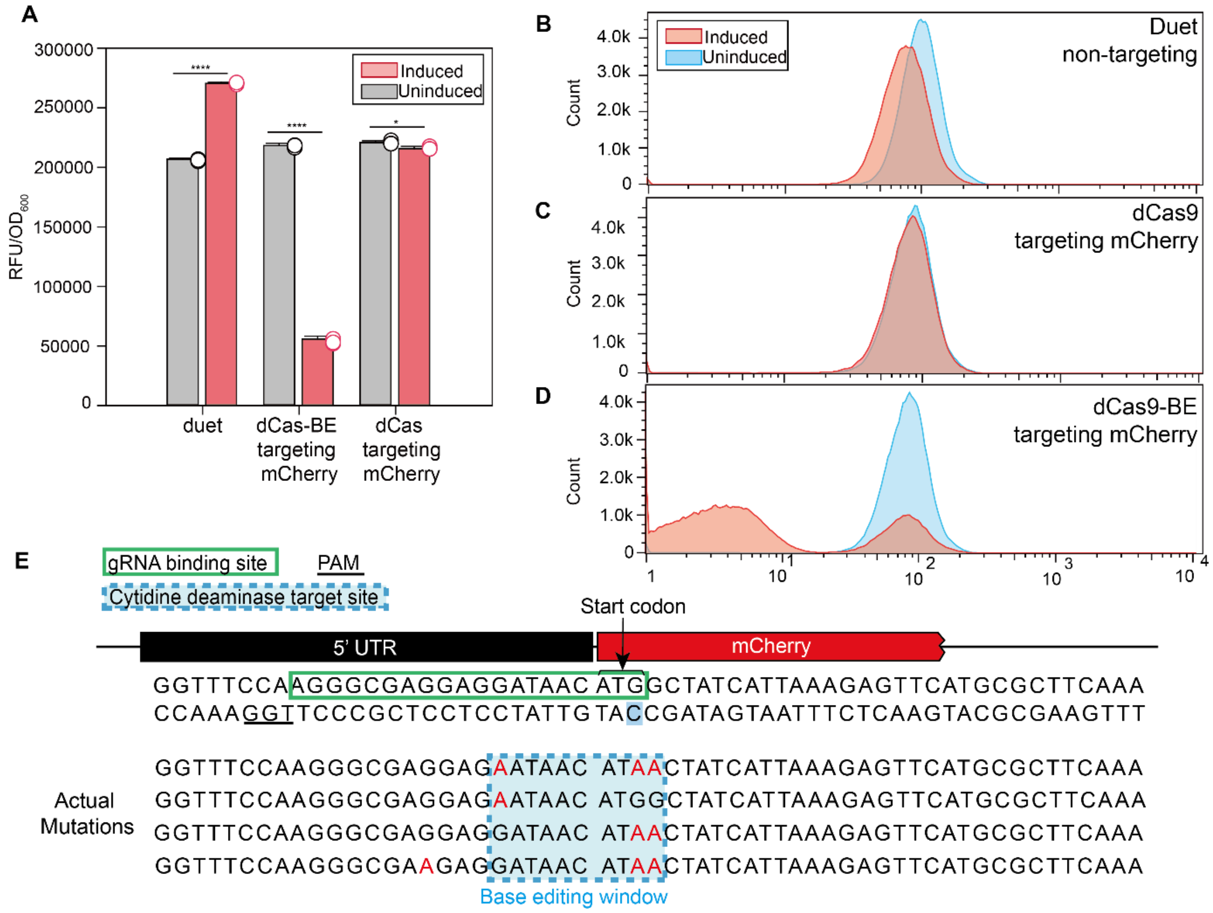

Fig. S3 Functional validation of base-editing-mediated reporter repression using a 5'UTR-proximal cytidine target. (a) Quantification of *mCherry* fluorescence (RFU/OD_{600}) under induced and uninduced conditions after a 16-hour overnight recovery culture without inducers. Strains harboring pDuet(control) vector, BE-*mCherry* (editing), and CE-*mcherry* (binding only) were tested. Bars represent mean values. The error bars represent the standard deviation from the biologically independent cell cultures (n = 3), and the white dots indicate the actual data points. The P-value of each strain's dataset was determined by the two-tailed Student's t-test compared to the dataset of the non-induced condition. (b-d) Flow cytometry histograms showing *mCherry* fluorescence expressing cells' distributions in induced (red) and uninduced (blue) conditions. Non-targeting controls (b) and interference-based inhibition system (c) exhibited minimal shift upon induction, whereas the editing *mCherry* system (d) resulted in a marked reduction in fluorescence following induction. (e) Sanger sequencing of the sorted 1% left-armed population after induction in editing condition. Expected base editing window (-16 ~ -19) is marked in light-blue and underlined GGT indicates the recognizable PAM sequence of the circuit. To verify the population that recovered target gene expression after transient induction, cells were collected after 8-hour induction, followed by a recovery culture for 16 hours in inducer-free media. Approximately 1×10^5 events were acquired. The lowest 1% of the fluorescence intensity distribution (1,000 events out of 100,000 total events) was sorted for downstream Sanger sequencing.

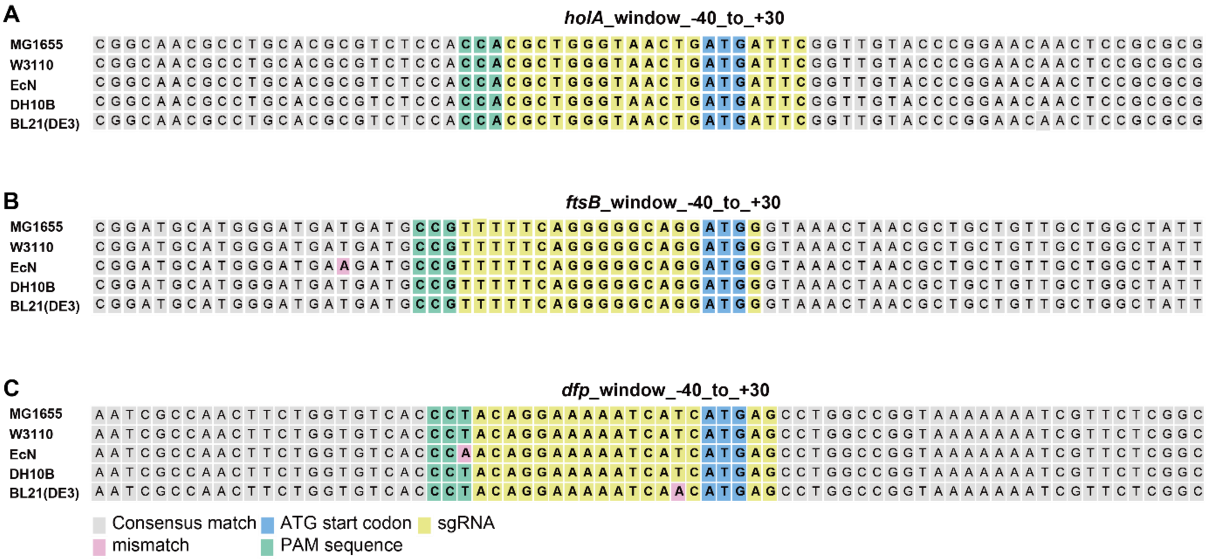

Fig. S4 Conservation of the start-codon-targeting window across representative *E.coli* strains. Sequence alignment of genomic regions (-40 to +30) spanning the editing window (-16 to -19) for three selected essential genes (*holA, ftsB,* and *dfp*) across representative *Escherichia coli* strains. The start codon is highlighted in blue, and the protospacer adjacent motif (PAM, NGG) is indicated in teal. The 20-nt sgRNA protospacer sequence is shown in yellow, with the effective editing window indicated within the protospacer. Conserved nucleotides are displayed in grey, and mismatches are marked in pink. The target sites selected for base editing were fully conserved across the examined strains, supporting the potential cross-strain portability of the start-codon-targeting containment strategy.

References

1. Doench, J.G., Fusi, N., Sullender, M., Hegde, M., Vaimberg, E.W., Donovan, K.F., Smith, I., Tothova, Z., Wilen, C. and Orchard, R. (2016) Optimized sgRNA design to maximize activity and minimize off-target effects of CRISPR-Cas9. *Nature biotechnology*, **34**, 184–191.

2. Moreno-Mateos, M.A., Vejnar, C.E., Beaudoin, J.-D., Fernandez, J.P., Mis, E.K., Khokha, M.K. and Giraldez, A.J. (2015) CRISPRscan: designing highly efficient sgRNAs for CRISPR-Cas9 targeting in vivo. *Nature methods*, **12**, 982–988.
